# Supplementary material for: Heat Treatment of Seeds to Control Invasive Common Ragweed (Ambrosia artemisiifolia), Narrow-Leaved Ragwort (Senecio inaequidens) and Giant Hogweed (Heracleum mantegazzianum)
Source: Plants (Basel). 2024 Jan 23;13(3):341. doi: 10.3390/plants13030341 (PMC10856870; doi:10.3390/plants13030341)
Supplement: Supplementary file 1 [file plants-13-00341-s001.zip › plants-2810306-supplementary.pdf]

Table S1. Survival rates (%) of seeds of common ragweed: a) (left columns): seeds at dry or wet condition, and protected or unprotected, exposed to different dry heat treatment temperatures (60, 70, 80, 90, or 100 °C) for 0.5, 1, 3, 6, 12, 24, or 48 hours; b) (upper right columns): wet or dry seeds treated with hot steam or hot water (both at 90 °C) for 0.5, 1, 5, 10, 20, or 40 minutes; c) (lower right columns) seeds treated in the field with hot foam for 5 minutes unprotected (lying on the soil surface), covered with vegetation and leaf litter, or buried 1 cm in soil n = 100* seeds per factor combination; 100 % mortification is marked in grey

Treatment temperature

DRY SEEDS unprotected	100°C	90°C	80°C	70°C	60°C
untreated control	100.0				
Treatment time					
0.5 hours	37.4	40.0	88.0	94.9	97.0
1 hour	18.6	26.8	67.0	96.0	94.0
3 hours	23.0	48.0	63.0	95.9	89.0
6 hours	26.3	28.3	73.0	91.9	86.7
12 hours	32.0	22.0	84.8	92.0	79.0
24 hours	4.0	13.0	79.0	88.0	96.0
48 hours	1.2	16.0	65.0	89.9	97.0

DRY SEEDS	Hot steam	Hot water
untreated control	98.9	
Treatment time		
0.5 minutes	100.0	6.1
1 minute	10.3	3.0
5 minutes	0.0	0.0
10 minutes	0.0	0.0
20 minutes	0.0	0.0
40 minutes	0.0	0.0

DRY SEEDS protected	100°C	90°C	80°C	70°C	60°C
untreated control	99.0				
Treatment time					
0.5 hours	92.9	86.9	94.9	94.7	97.8
1 hour	92.0	78.4	91.9	94.8	92.6
3 hours	78.5	49.5	87.2	86.5	89.6
6 hours	53.9	43.8	92.6	87.2	89.0
12 hours	33.3	51.0	82.1	84.4	92.8
24 hours	28.4	53.6	94.6	81.9	96.7
48 hours	14.9	40.4	91.3	73.2	95.8

DRY SEEDS	Hot foam
untreated control	94.9
Treatment	
unprotected	75.0
vegetation cover	88.0
in 1 cm soil depth	75.0

WET SEEDS unprotected	100°C	90°C	80°C	70°C	60°C
untreated control	97.0				
Treatment time					
0.5 hours	48.5	71.1	87.0	96.9	95.0
1 hour	6.7	7.1	29.2	75.8	87.0
3 hours	1.1	3.0	18.0	47.5	59.8
6 hours	2.2	4.2	7.1	14.1	66.3
12 hours	0.0	0.0	4.2	12.2	44.2
24 hours	0.0	0.0	0.0	11.1	36.4
48 hours	0.0	0.0	0.0	13.5	55.0

WET SEEDS	Hot steam	Hot water
untreated control	98.9	
Treatment time		
0.5 minutes	85.9	2.0
1 minute	2.0	0.0
5 minutes	0.0	0.0
10 minutes	0.0	0.0
20 minutes	0.0	0.0
40 minutes	0.0	0.0

WET SEEDS protected	100°C	90°C	80°C	70°C	60°C
untreated control	86.0				
Treatment time					
0.5 hours	80.4	94.7	95.8	94.7	91.6
1 hour	89.5	77.6	90.8	77.2	90.4
3 hours	41.8	51.0	85.4	91.5	89.8
6 hours	3.1	50.5	64.9	76.3	96.9
12 hours	0.0	43.9	70.9	73.7	85.3
24 hours	0.0	36.1	61.1	59.0	84.4
48 hours	0.0	15.5	44.8	64.2	84.5

WET SEEDS	Hot foam
untreated control	97.0
Treatment	
unprotected	39.5
vegetation cover	43.5
in 1 cm soil depth	0.0

*Seeds which were visibly intact but turned out to be empty when opened (no embryo developed) were excluded from the results as the viability of these seeds was not affected by the heat treatments; total number of seeds per factor combination varies between 98 and 100 seeds

Table S2. Survival rate (%) of dry and wet seeds of narrow-leaved ragwort (protected and unprotected) after exposure to different treatment temperatures (60, 70, 80, 90, 100°C) for 0.5, 1, 3, 6, 12, 24, and 48 hours, after treatment with hot steam and hot water (both. 90°C) for 0.5, 1, 5, 10, 20, and 40 minutes, and after treatment with hot foam for 5 minutes unprotected (lying on the soil surface), covered with surrounding vegetation or buried 1 cm in soil n= 100* seeds per factor combination; 100 % mortification is marked in grey

	Treatment temperature				
**DRY SEEDS unprotected**	**100°C**	**90°C**	**80°C**	**70°C**	**60°C**
**untreated control**	**92.0**				
Treatment time					
**0.5 hours**	67.7	84.0	89.0	90.0	86.0
**1 hour**	71.3	88.0	90.0	100.0	85.0
**3 hours**	82.0	94.1	90.0	96.0	85.6
**6 hours**	49.0	81.0	90.1	92.0	85.0
**12 hours**	83.2	80.0	84.0	99.0	84.3
**24 hours**	49.0	75.0	91.0	95.0	91.9
**48 hours**	22.0	66.3	82.0	96.0	88.0

**DRY SEEDS**	**Hot steam**	**Hot water**
**untreated control**	**98.0**	
Treatment time		
**0.5 minutes**	100.0	0.0
**1 minute**	33.3	0.0
**5 minutes**	22.0	0.0
**10 minutes**	1.0	0.0
**20 minutes**	0.0	0.0
**40 minutes**	0.0	0.0

**DRY SEEDS protected**	**100°C**	**90°C**	**80°C**	**70°C**	**60°C**
**untreated control**	**97.0**				
Treatment time					
**0.5 hours**	99.0	94.0	87.1	100.0	91.0
**1 hour**	94.0	91.0	91.0	97.0	85.7
**3 hours**	92.0	88.0	84.0	97.0	88.0
**6 hours**	95.0	84.0	93.0	94.0	92.0
**12 hours**	97.0	86.1	93.0	95.0	85.1
**24 hours**	76.0	83.0	83.0	98.0	88.9
**48 hours**	39.0	68.0	88.0	93.0	86.1

**DRY SEEDS**	**Hot foam**
**untreated control**	**95.0**
Treatment	
**unprotected**	95.0
**vegetation cover**	0.0
**in 1 cm soil depth**	0.0

**WET SEEDS unprotected**	**100°C**	**90°C**	**80°C**	**70°C**	**60°C**
**untreated control**	**96.0**				
Treatment time					
**0.5 hours**	0.0	0.0	0.0	72.2	94.0
**1 hour**	0.0	0.0	0.0	46.0	82.0
**3 hours**	0.0	0.0	0.0	30.0	98.0
**6 hours**	0.0	0.0	0.0	14.9	97.0
**12 hours**	0.0	0.0	0.0	56.0	90.0
**24 hours**	0.0	0.0	0.0	40.4	95.9
**48 hours**	0.0	0.0	0.0	56.0	88.7

**WET SEEDS**	**Hot steam**	**Hot water**
**untreated control**	**97.0**	
Treatment time		
**0.5 minutes**	15.0	0.0
**1 minute**	27.7	0.0
**5 minutes**	0.0	0.0
**10 minutes**	0.0	0.0
**20 minutes**	0.0	0.0
**40 minutes**	0.0	0.0

**WET SEEDS protected**	**100°C**	**90°C**	**80°C**	**70°C**	**60°C**
**untreated control**	**92.0**				
Treatment time					
**0.5 hours**	0.0	0.0	28.0	93.0	84.0
**1 hour**	0.0	0.0	29.0	77.6	84.4
**3 hours**	0.0	0.0	38.4	70.0	94.0
**6 hours**	0.0	0.0	45.5	66.0	96.0
**12 hours**	0.0	0.0	23.0	52.0	86.6
**24 hours**	0.0	0.0	29.2	66.0	82.0
**48 hours**	0.0	0.0	37.4	79.2	88.0

**WET SEEDS**	**Hot foam**
**untreated control**	**97.0**
Treatment	
**unprotected**	49.0
**vegetation cover**	0.0
**in 1 cm soil depth**	0.0

*Seeds which were visibly intact but turned out to be empty when opened (no embryo developed) were excluded from the results as the viability of these seeds was not affected by the heat treatments; total number of seeds per factor combination varies between 97 and 100 seeds

Table S3. Survival rate (%) of dry and wet seeds of giant hogweed (protected and unprotected) after exposure to different treatment temperatures (60, 70, 80, 90, 100°C) for 0.5, 1, 3, 6, 12, 24, and 48 hours, after treatment with hot steam and hot water (both. 90°C) for 0.5, 1, 5, 10, 20, and 40 minutes, and after treatment with hot foam for 5 minutes unprotected (lying on the soil surface), covered with surrounding vegetation or buried 1 cm in soil n= 100* seeds per factor combination; 100 % mortification is marked in grey

	Treatment temperature				
**DRY SEEDS unprotected**	**100°C**	**90°C**	**80°C**	**70°C**	**60°C**
**untreated control**	**85.0**				
Treatment time					
**0.5 hours**	67.0	76.0	82.0	84.7	91.9
**1 hour**	51.0	72.7	79.8	79.8	89.8
**3 hours**	35.0	62.2	72.3	75.8	88.8
**6 hours**	12.1	60.0	75.3	83.7	82.8
**12 hours**	0.0	47.0	36.4	79.6	81.0
**24 hours**	0.0	7.0	36.0	79.4	78.0
**48 hours**	0.0	0.0	33.0	64.3	72.0

**DRY SEEDS**	**Hot steam**	**Hot water**
**untreated control**	**91.0**	
Treatment time		
**0.5 minutes**	84.9	0.0
**1 minute**	56.1	0.0
**5 minutes**	0.0	0.0
**10 minutes**	0.0	0.0
**20 minutes**	0.0	0.0
**40 minutes**	0.0	0.0

**DRY SEEDS protected**	**100°C**	**90°C**	**80°C**	**70°C**	**60°C**
**untreated control**	**87.0**				
Treatment time					
**0.5 hours**	73.0	85.0	85.9	85.7	94.9
**1 hour**	54.1	84.0	83.8	85.9	92.9
**3 hours**	43.0	75.0	76.0	79.6	84.9
**6 hours**	22.0	65.3	70.7	78.8	82.7
**12 hours**	0.0	59.2	63.6	77.8	80.6
**24 hours**	0.0	8.0	67.7	72.5	75.5
**48 hours**	0.0	0.0	46.5	62.6	68.3

**DRY SEEDS**	**Hot foam**
**untreated control**	**87.0**
Treatment	
**unprotected**	79.8
**vegetation cover**	31.0
**in 1 cm soil depth**	0.0

**WET SEEDS unprotected**	**100°C**	**90°C**	**80°C**	**70°C**	**60°C**
**untreated control**	**86.0**				
Treatment time					
**0.5 hours**	0.0	13.0	71.0	67.0	85.0
**1 hour**	0.0	0.0	65.7	46.9	83.8
**3 hours**	0.0	0.0	0.0	53.0	70.7
**6 hours**	0.0	0.0	0.0	4.1	76.5
**12 hours**	0.0	0.0	0.0	0.0	73.3
**24 hours**	0.0	0.0	0.0	0.0	60.6
**48 hours**	0.0	0.0	0.0	0.0	61.6

**WET SEEDS**	**Hot steam**	**Hot water**
**untreated control**	**86.0**	
Treatment time		
**0.5 minutes**	0.0	0.0
**1 minute**	0.0	0.0
**5 minutes**	0.0	0.0
**10 minutes**	0.0	0.0
**20 minutes**	0.0	0.0
**40 minutes**	0.0	0.0

**WET SEEDS protected**	**100°C**	**90°C**	**80°C**	**70°C**	**60°C**
**untreated control**	**86.0**				
Treatment time					
**0.5 hours**	76.0	78.0	71.0	91.8	91.9
**1 hour**	0.0	25.3	65.0	89.8	88.0
**3 hours**	0.0	0.0	0.0	30.3	87.9
**6 hours**	0.0	0.0	0.0	28.6	74.8
**12 hours**	0.0	0.0	0.0	31.6	71.4
**24 hours**	0.0	0.0	0.0	31.0	72.7
**48 hours**	0.0	0.0	0.0	5.1	67.0

**WET SEEDS**	**Hot foam**
**untreated control**	**86.0**
Treatment	
**unprotected**	80.0
**vegetation cover**	0.0
**in 1 cm soil depth**	0.0

*Seeds which were visibly intact but turned out to be empty when opened (no embryo developed) were excluded from the results as the viability of these seeds was not affected by the heat treatments; total number of seeds per factor combination varies between 99 and 100 seeds

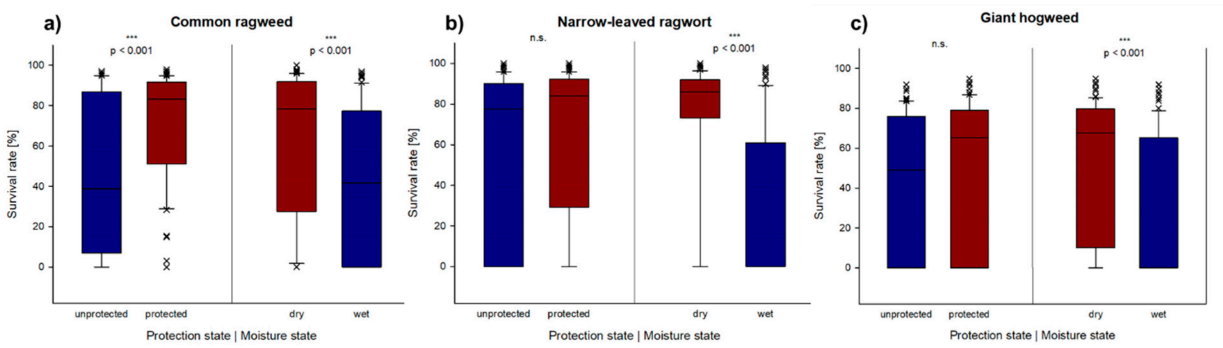

Figure S1: Seed survival rate (%) after dry air heat treatment in lab of a) common ragweed, b) narrow-leaved ragwort, and c) giant hogweed in relation to the seed protection state (unprotected = air heated; protected = incorporated in soil) and to the moisture
